# Dynamic changes in brain structure in patients with post-traumatic stress disorder after motor vehicle accident: A voxel-based morphometry-based follow-up study

**DOI:** 10.3389/fpsyg.2022.1018276

**Published:** 2022-10-06

**Authors:** Luodong Yang, Haohao Li, Yao Meng, Yan Shi, Anxin Ge, Guiqing Zhang, Chaomeng Liu

**Affiliations:** ^1^Shihezi University School of Medicine, Shihezi, China; ^2^First Affiliated Hospital of Shihezi University School of Medicine, Shihezi, China; ^3^Beijing Key Laboratory of Mental Disorders, National Clinical Research Center for Mental Disorders and National Center for Mental Disorders, Beijing Anding Hospital, Capital Medical University, Beijing, China; ^4^Advanced Innovation Center for Human Brain Protection, Capital Medical University, Beijing, China

**Keywords:** PTSD, fMRI, social support, coping style, motor vehicle accident

## Abstract

**Objectives:**

To investigate the dynamic changes of emotional and memory-related brain regions in post-traumatic stress disorder (PTSD) patients and trauma-exposed subjects, who experienced motor vehicle accident (MVA).

**Materials and methods:**

Functional Magnetic Resonance imaging (fMRI) and general data were collected from trauma victims who had experienced MVA within 2 days, and their social support and coping style were evaluated. The PTSD Checklist for Diagnostic and Statistical Manual of Mental Disorders-Fifth Edition (PCL-5) is used for screening and diagnosis. Subsequently, 17 PTSD patients and 23 car accident trauma-exposed individuals completed a second fMRI scan at 2 months. Data were analyzed by using voxel-based morphometry (VBM) to examine the volume changes of relevant brain regions. Correlation analysis was used to assess the correlation between the regions of interest (ROIs) and the total scores on the clinical scales. Subsequently, the relationship between the total PCL-5 scores and the individual dimensions of the Simplified Coping Style Questionnaire (SCSQ) and the Social Support Rate Scale (SSRS) was studied.

**Results:**

In comparison with the control group, the results showed a reduction in right SFG volume in the PTSD group at 2 months. Similarly, a comparison within the PTSD group revealed a reduction in the left STG volume at 2 months. Compared with the control group, PTSD patients showed a more negative coping style and worse performance in objective and subjective support. In addition, the total PCL-5 scores were negatively associated with positive coping, objective support, and subjective support.

**Conclusion:**

The occurrence of PTSD may be related to reduced volume of the right SFG and left STG, and that patients with PTSD receive less social support and tend to cope in a negative manner in the face of stressful events. These results suggest that within 2 months of the MVA, changes in gray matter volume have occurred in some brain regions of those suffering from PTSD. We believe the results of our study will provide useful insights into the neuropsychological mechanisms underlying PTSD.

## Introduction

Post-traumatic stress disorder (PTSD) refers to a mental disorder frequently occurring after experiencing or witnessing a traumatic event (North et al., 2016). Symptoms of PSTD include intrusive memory, negative cognition, hypervigilance, and avoidance. With the increasing popularity of vehicles, PTSD caused by traffic accidents has emerged as one of the most common mental disorders among the survivors of adult road traffic accidents. The incidence of PTSD after traffic accidents ranges from 6 to 45% (Heron-Delaney et al., 2013). Studies have demonstrated that the prevalence of PTSD can reach 23.1% 3 months after a traffic accident (Ehlers et al., 1998). PTSD can affect the quality of life, resulting in psychological, physical, and cognitive changes (Yehuda et al., 1995), consequently increasing the risk of suicide (Pietrzak et al., 2011).

Several previous neuroimaging studies have demonstrated that PTSD is related to abnormalities in the structure of the brain. Understanding the pathological changes in the structure of the brain may reveal the neuropsychological mechanism underlying PTSD. Recently, functional magnetic resonance imaging (fMRI) has allowed rapid development in studying the neurological function and structure of the brain, resulting in good prospects for investigating the neurophysiological mechanisms underlying PTSD.

Studies have reported that individuals with PTSD have volume changes in the areas of the brain’s gray matter, including the amygdala, anterior cingulate cortex, hippocampus, parahippocampal gyrus, prefrontal cortex, temporal cortex, and insula (Nemeroff et al., 2006; Francati et al., 2007; Morey et al., 2012; Kühn and Gallinat, 2013; McLaughlin et al., 2014; Meng et al., 2014; Kennis et al., 2015). According to a theory, the reduced gray matter volume reflects a preexisting vulnerability that increases the propensity to develop PTSD. A study on identical twins reported that a previously smaller hippocampus was the risk factor for developing stress-related psychopathology. In addition, disease severity was negatively associated with hippocampal volume in patients with PTSD (Gilbertson et al., 2002).

Voxel-based morphometry (VBM) can be adopted to evaluate the brain volume on a voxel-by-voxel basis and study the morphological changes in the structure of the brain non-invasively without specifying the region of interest in advance. VBM technology has been successfully used in several diseases, including depression (Peng et al., 2011), Parkinson’s disease (Pickut et al., 2013), epilepsy (Bernasconi et al., 2004), and Alzheimer’s disease (Baxter et al., 2006). A previous study using VBM compared 12 fire-induced patients with PTSD with 12 fire-affected controls and reported that PTSD patients had reduced gray matter volume in three brain regions; namely, the left hippocampus left anterior cingulate cortex, and bilateral insular cortex (Chen et al., 2006). In another VBM study, a decrease in the overall hippocampal gray matter volume was discovered in 23 patients with PTSD compared with 17 healthy individuals (Emdad et al., 2006). In addition, in a VBM study, 25 patients with PTSD showed reduced gray matter volumes, particularly in the frontal and occipital lobes, compared with 25 healthy controls (Tavanti et al., 2012). However, these studies did not longitudinally compare the differences in the brain regions between the two groups.

Therefore, the focus of the current work was to dynamically identify the changes in the volume of emotional and memory-related brain regions in patients with PTSD after MVA, starting from the traumatic events experienced by patients. Longitudinal and cross-sectional comparisons were performed between the trauma-exposed and PTSD groups. We predicted that compared with controls, PTSD patients after traumatic exposure had reduced gray matter volume in emotional and memory-related areas of the brain. In addition, we hypothesized that the severity of symptoms and voxel changes in the related brain regions were associated with social support and coping styles. We believe our study will provide insights into the neuropsychological mechanisms underlying PTSD.

## Materials and methods

### Participants

**FIGURE 1:**
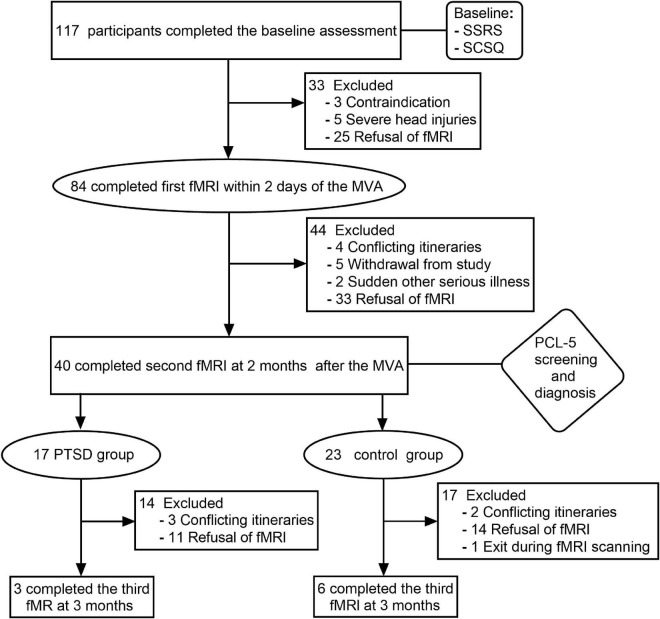
Flowchart.

Participants were volunteers who were recruited between December 2018 and November 2019. All participants were from the emergency departments of two tertiary care hospitals in Shihezi and had suffered injuries in a car accident. During the first assessment, participants’ general clinical data were collected, and a baseline assessment of their psychological status was made using the Simple Coping Style Disorder questionnaire (SCSQ) and the Social Support Rating Scale (SSRS). In total, 117 right-handed subjects (60 males and 57 females) were enrolled in the study after exposure to MVA and after completing baseline assessments. At the end of the evaluation, 84 participants consented and completed their first fMRI scan. All baseline assessments and fMRI scans were completed within 2 days after the accident. Then PCL-5 was used to assess the symptoms of PTSD at 1 month, 2 months, and 3 months after the accident. A PCL-5 score greater than 33 was considered a positive criterion to screen PTSD symptoms. Those exposed to traumatic events and who were screened as positive were subsequently diagnosed with PTSD by two or more attending physicians. Of the 117 participants, 20 were diagnosed with PTSD within 3 months. In addition, 97 participants had no PTSD. Subsequently, 17 PTSD patients and 23 car accident trauma-exposed individuals completed a second fMRI scan at 2 months, of which only 3 of the PTSD patients and 6 of the control group participants completed a third fMRI scan 3 months after the accident (Figure 1). Finally, all patients undergoing PTSD received conventional psychological or medical treatment.

The exclusion criteria were:

(1) Age < 18 years or > 65 years;
(2) Severe head injuries (abnormalities on conventional MRI, neurological abnormality during emergency department evaluation, or loss of consciousness longer than several seconds during the accident based on self-report by the participant);
(3) Those with a history of neurological disease or mental disorder;
(4) Patients with previous brain trauma and severe somatic symptoms;
(5) Drug abuse, alcohol addiction, or recent use of drugs that could affect brain function;
(6) Women during pregnancy and breastfeeding;
(7) MRI scans are contraindicated (metal placement, severe claustrophobia).

The image data were collected and analyzed by two experienced radiologists who were unaware of the enrollment images of the two groups. This study was approved by the Ethics Committee of the Shihezi University School of Medicine. Written informed consent was obtained from each subject before participation. All procedures followed the guidelines of the ethics agency.

### Assessment of post-traumatic stress disorder and mental status

#### The post-traumatic stress disorder checklist for diagnostic and statistical manual of mental disorders-fifth edition (PCL-5)

The PCL-5, based on the Diagnostic and Statistical Manual of Mental Disorders (Bovin et al., 2016), 5th edition, published by the American Psychiatric Association, is a measurement tool to evaluate the presence and severity of PTSD. On a 5-point scale of 20 items (0 = “not at all” to 4 = “extreme”), the rating includes four core symptom clusters, following the DSM-5 diagnostic rules. PCL-5 is an international universal scale with high reliability and validity. A total score of PCL-5 ≥ 33 served as the positive criterion for screening PTSD symptoms, and a higher score was related to a higher likelihood of PTSD.

#### The Simple Coping Style Questionnaire

The SCSQ is a 20-item self-report scale that is used to measure an individual’s coping style (Xie, 1998). Using the scale developed by Ya’ning Xie (1998), SCSQ is classified into two subscales, namely, positive coping (12 items) and negative coping (8 items). Each item is assigned a score on a 4-point scale (0 = never, 1 = rarely, 2 = often, and 3 = always). A higher score for each subscale reflects the corresponding coping style level. Cronbach’s α values for positive coping and negative coping were 0.89 and 0.78, respectively. The scale has a high reliability and validity test level.

#### The Social Support Rating Scale

The SSRS is a three-level self-report scale with 10 items that are used to measure the degree of social support of an individual (Shuiyuan, 1994). The scale compiled by Shuiyuan Xiao in 1986 based on emergency research consists of 66 points. The SSRS is divided into three dimensions, i.e., objective support (3 items, 22 points), subjective support (4 items, 32 points), and useless support (3 items, 12 points). A higher score on each dimension relates to a higher level of social support. Cronbach’s α value of this scale was 0.808.

#### Adult psychological test analysis tool

The comprehensive psychological test software (adult psychological test System version 5.0) developed by the Shanghai Huicheng Company Limited was used to input and analyze the results of SSRS, SCSQ, and PCL-5.

### Magnetic resonance imaging data acquisition

The participants were scanned using a 3.0 T MRI machine (GE Discovery MR750 from GE, USA) in the imaging department of the First Affiliated Hospital of Shihezi University School of Medicine in a resting state MRI. Before the scan, participants were assisted to lie in a flat position, use earplugs to reduce noise, and were instructed to minimize head movement, remain awake, close their eyes, and breathe calmly. All participants underwent MRI scans with the same parameters, using T1 structural images and gradient echo-planar imaging (GRE-EPI). T1 structural imaging was used to detect brain lesions using the following parameters: repetition time (TR)/echo time (TE) = 8.2 ms/3.2 ms; matrix = 256 × 256; field-of-view = 256 mm × 256 mm; flip angle = 12°; slice thickness = 1.0 mm; number of layers = 148; The scan time is 4 min and 20 s. GRE-EPI sequence parameters were repeat time (TR)/echo time (TE) = 2,000 ms/30 ms; matrix = 64 × 64; field-of-view = 240 mm × 240 mm; flip angle = 90°; slice thickness = 4 mm; scan = 32 layers, layer spacing = 0 mm; scan time = 8 min and 20 s. It contains 245 time points. After the scan, participants were asked questions to assess how well the subjects cooperated.

### Magnetic resonance imaging data analysis of voxel-based morphometry

The data were analyzed by an optimized VBM implemented in SPM. The standard image data were prepared, pre-processed, and statistically analyzed using the VBM5 package in the Statistical Parametric Map SPM5^1^ toolbox in MatLab7.1. The major processing steps were: Using affine transformation, all original images were aligned to the Montreal Neurological Institute (MNI) standard template. Next, the aligned images were segmented into gray matter, white matter, cerebrospinal fluid, and non-cerebral voxels. The segmented gray matter images were corrected for volume changes using Jacobi determinants to compensate for the effects of spatial normalization. All normalized, segmented, and corrected gray matter images were smoothed with the 12 mm full width at half maximum (FWHM) Gaussian smoothing kernel.

### Statistical analysis

DPABI4.0 was used to statistically analyze the image results. A two-sample *t*-test was adopted to evaluate the differences in VBM values between PTSD and the control group at 2 days and 2 months. The Gaussian random field theory-based multiple comparison correction was further performed (GRF, voxel-wise *P* = 0.005, cluster-wise *P* = 0.05). Paired *t*-test was applied to evaluate the VBM values of PTSD and the control group before and after the correction Gaussian Random Field (GRF, voxel-wise *P* = 0.005, cluster-wise *P* = 0.05). Previous studies have reported that in comparison with Bonferroni correction and false discovery rate (FDR), GRF uses the spatial information of fMRI data to decrease the false-positive rates and enhance the statistical power (Gong et al., 2018). In the current work, data were statistically analyzed using SPSS 26.0 (SPSS, Chicago, IL, USA), with numerical variables denoted as mean ± standard deviation and categorical variables (gender) denoted as counts and percentages. A two-sample *t*-test was applied to evaluate the differences between PTSD patients and controls on age, education, PCL-5, SCSQ, and SSRS scores. *P* < 0.05 was regarded to be a statistically significant difference. Correlation analysis was used to assess the correlation between the regions of interest (ROIs) and the total scores on the clinical scales, i.e., PCL-5, SCSQ, and SSRS. Subsequently, the relationship between the total PCL-5 scores and the individual dimensions of SCSQ and SSRS was studied. For correlation analysis, Pearson’s correlation was used for both variables showing normal distribution, and Spearman’s correlation was used if they were non-normally distributed.

## Results

### Demographic data and measurement of the psychological stress response

**FIGURE 2:**
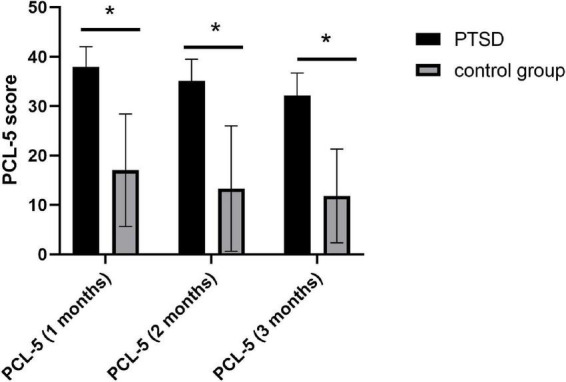
Changes in PCL-5 scores measured at 1, 2, and 3 months; *P < 0.001.

In total, 17 patients with PTSD and 23 participants in the control group after MVA were finally included in this study. The age of patients with PTSD and the control group were 43.3 ± 12.1 years and 41.2 ± 13.1 years, respectively. No significant differences were observed in age, gender, education, and support for the useless support of SSRS between the two groups; however, we detected notable differences in the objective and subjective support of SSRS, positive and negative coping of SCSQ, and three measurement scores of PCL-5 (Table 1), *P* < 0.05. The scores of three measurements of PCL-5 showed a downward trend over time (Figure 2).

**TABLE 1:** Demographic data and psychological stress response scores at baseline and PCL-5 scores at 3 months of follow-up in PTSD patients and controls after motor vehicle accident.

Characteristic	PTSD group (*N* = 17)	Control group (*N* = 23)	*t*-value	*P-value*
Age mean (SD) (y)	45.3 ± 12.1	41.2 ± 13.1	1.008	0.320
Sex*. No. (%)				
Male	5 (29.4)	13 (56.5)		
Female	12 (70.6)	10 (43.5)		
Education mean (SD)(y)	11.5 ± 3.8	12.3 ± 3.8	–0.693	0.492
**SCSQ score, mean (SD)**				
Positive coping	15.5 ± 5.1	22.3 ± 4.9	–4.316	0.001*
Negative coping	15.3 ± 3.0	10.5 ± 4.8	3.626	0.001*
**SSRS score, mean (SD)**				
Objective support	14.6 ± 2.6	25.7 ± 6.0	–7.956	0.001*
Subjective support	7.8 ± 2.0	11.0 ± 4.3	–2.790	0.008*
Useless support	6.0 ± 2.0	6.5 ± 2.6	–0.636	0.528
PCL-5 (1 months post-accident)	37.9 ± 4.1	17.0 ± 11.4	8.121	0.001*
PCL-5 (2 months post-accident)	35.1 ± 4.4	13.3 ± 12.7	7.640	0.001*
PCL-5 (3 months post-accident)	32.1 ± 4.6	11.8 ± 9.5	8.925	0.001*

PTSD, post-traumatic stress disorder; PCL-5, the PTSD Checklist for DSM-5; SCSQ, the Simplified Coping Style Questionnaire; SSRS, the Social Support Rate Scale.

*For gender composition, X^2^ = 2.903 and *P* = 0.088.

*p < 0.001.

### Changes in related brain regions between post-traumatic stress disorder and control groups

**FIGURE 3:**
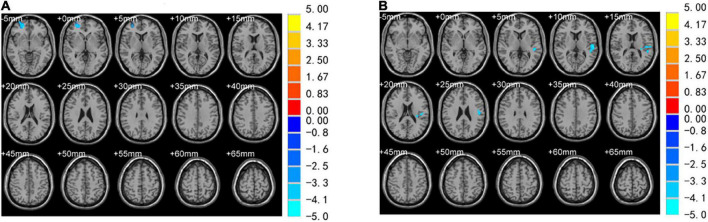
Brain regions with significantly different VBM values. **(A)** Volume reduction in the right superior frontal gyrus (SFG) was observed in PTSD and control group for 2 months. **(B)** The volume of the left superior temporal gyrus (STG) was reduced in the PTSD group at 2day and 2 months.

No differences were observed in the brain regions between PTSD and control groups when using VBM at baseline. A comparison between groups at 2 months showed that the volume of the right SFG (Brodmann area [BA] 10) decreased (Table 2 and Figure 3A). In the PTSD group, the volume of the left STG (Brodmann area [BA] 22) decreased at 2 months (Table 3 and Figure 3B). Compared to the baseline, no abnormally different brain areas were found in the control group at 2 months.

**TABLE 2:** Brain regions with significantly different VBM values of the PTSD group and the control group at 2 months.

Brain regions of peak coordinates	MNI coordinates			BA	Number of voxels	*t*-value
				
	*X*	*Y*	*Z*			
Right superior frontal gyrus	18	51	−3	10	1040	−4.9351

MNI, Montreal neurological institute; BA, Brodmann’s area.

**TABLE 3:** Brain regions with significantly different VBM values of the PTSD at 2 days and 2 months.

Brain regions of peak coordinates	MNI coordinates			BA	Number of voxels	*t*-value
				
	*X*	*Y*	*Z*			
Left superior temporal gyrus	−48	−15	22.5	22	1094	−5.409

MNI, Montreal neurological institute; BA, Brodmann’s area.

### Correlation analysis

**FIGURE 4:**
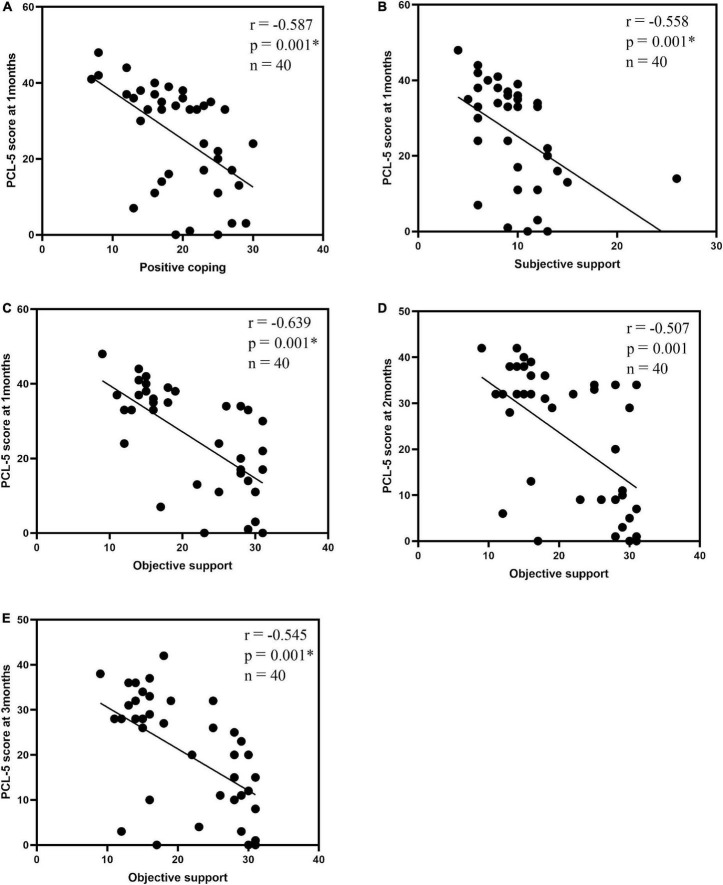
Correlation analysis| between the total PCL-5 score and the SCSQ, SSRS dimensions: 1-month PCL-5 scores and positive coping **(A)**, subjective support **(B)**; objective support and 1-month PCL-5 scores **(C)**, 2-month PCL-5 scores **(D)**, 3-month PCL-5 scores **(E)**. P < 0.003 was considered a statistically significant difference. *p < 0.001.

In the PTSD group, correlation analyses were performed using the differential ROI values of the left STG with PCL-5, SCSQ, and SSRS. The differences were statistically insignificant (*p* < 0.01, *n* = 17) using Bonferroni-based correction for multiple comparisons. Associations between the total PCL-5 score and SCSQ and SSRS dimensions were analyzed for PTSD and control groups. Using Bonferroni-based multiple comparison correction, *p* < 0.003 was considered statistically significant, 1-month PCL-5 scores were negatively correlated with positive coping and subjective support (*r* = –0.587, *p* = 0.001*, *n* = 40; *r* = –0.558, *p* = 0.001*, *n* = 40), and three-time PCL-5 scores showed a negative correlation with objective support (*r* = –0.639, *p* = 0.001*, *n* = 40; *r* = –0.507, *p* = 0.001, *n* = 40; *r* = –0.545, *p* = 0.001*, *n* = 40; Table 4 and Figure 4).

**TABLE 4:** Correlation between 3 PCL-5 scores and social support, coping style scale scores.

	PCL-5 (1 month)		PCL-5 (2 month)		PCL-5 (3 month)	
			
	*R*	*p*	*r*	*p*	*r*	*p*
**SCSQ**						
Positive coping	−0.587	0.001*	−0.414	0.008	−0.403	0.010
Negative coping	0.342	0.031	0.388	0.013	0.424	0.006
**SSRS**						
Objective support	−0.639	0.001*	−0.507	0.001	−0.545	0.001*
Subjective support	−0.558	0.001*	−0.300	0.060	−0.299	0.061
Useless support	−0.143	0.380	−0.222	0.169	−0.122	0.455

PCL-5, the PTSD Checklist for DSM-5; SCSQ, the Simplified Coping Style Questionnaire; SSRS, the Social Support Rate Scale.

*p < 0.001.

## Discussion

We used VBM to dynamically observe changes in memory and related brain volume in PTSD patients after MVA. In comparison with the control group, the results showed a reduction in right SFG volume in the PTSD group at 2 months. Similarly, a comparison within the PTSD group revealed a reduction in the left STG volume at 2 months. Compared with the control group, PTSD patients showed a more negative coping style and worse performance in objective and subjective support. In addition, the total PCL-5 scores were negatively associated with positive coping, objective support, and subjective support.

Next, a reduction in the volume of the right SFG was detected in patients with PTSD after MVA, which is consistent with the findings of several previous studies (Wang et al., 2016; O’Doherty et al., 2017). A meta-analysis reported reduced gray matter volumes in the left hippocampus, left middle frontal gyrus, as well as right SFG in the PTSD group compared to the trauma-exposed group (Li et al., 2014). Therefore, we hypothesize that right SFG injury is prevalent in PTSD. The medial surface of the SFG forms the medial aspect of the prefrontal cortex, and certain scholars believe that the medial prefrontal cortex is an important functional center of the Default Mode Network (DMN) (Kunimatsu et al., 2020). A study on the central executive network (CEN), DMN, and the salience network (SN) reported that PTSD patients had reduced frontal gyrus connectivity in the DMN bilaterally (Liu et al., 2017). Similarly, a previous (regional homogeneity) REHO study discovered that patients with PTSD displayed enhanced REHO values in the left inferior parietal lobule and right SFG and that right superior frontal gyrus REHO values were associated with the activation of negative emotions (Yin et al., 2012). At 2 days after MVA, no difference in the brain area was reported between PTSD and control groups; however, 2 months later, a decrease in the volume of gray matter in the right SFG of the PTSD group suggested that the development of PTSD was not attributed to a congenital defect in brain function. Thus, the traumatic environment contributes to PTSD.

STG is associated with speech perception and is involved in the higher-order auditory processing of speech. The literature on superior temporal gyrus in PTSD is scarce (Mesgarani et al., 2014). A large multi-center study of brain structure reported a highly reduced volume of the left STG in the PTSD group than in the control group (Wang et al., 2021). Studies on functional connectivity reported reduced functional connectivity of the STG to the basolateral amygdala in the trauma-exposed and PTSD groups compared with the healthy group (Liu et al., 2021). In addition, a study of individuals exposed to life stress using REHO reported prolonged exposure to higher levels of life stress, resulting in reduced REHO in STG and a negative correlation of REHO values with the severity of stress (Philip et al., 2013). In this study, the PTSD group showed a reduced volume of the left STG after 2 months of post-traumatic experience, consistent with the results of previous studies.

In a meta-analysis, compared to controls, a reduction in the gray matter volume in the left insular cortex and right anterior cingulate cortex of PTSD caused by natural disasters was reported. The unnatural disasters resulted in less gray matter volume in the left medial frontal gyrus in the PTSD group compared with the control group (Meng et al., 2014). Another study reported cortical thinning of the left parietal lobe, right inferior frontal gyrus, as well as right parahippocampal gyrus in the PTSD group that had experienced a mining accident compared with the group that had not experienced PTSD (Liu et al., 2012). In addition, a reduction in the gray matter volume in the left hippocampus left anterior cingulate, and bilateral insula cortices were reported in one post-fire PTSD group compared with the control group (Chen et al., 2006). In the present study, a reduction in the volume of the right SFG and left STG was recorded in patients with PTSD after MVA. This finding suggests that different types of trauma cause PTSD and that their differential brain regions could differ.

Regarding the neuropsychological mechanism of PTSD, certain scholars believe that symptoms of flashbacks, nightmares, and compulsion to recall traumatic scenes are attributed to reduced cognitive control, such as reduced volume or diminished function of gray matter in the anterior cingulate gyrus and brainstem (Elzinga and Bremner, 2002). Other studies have reported that one of the pathogenetic theories of PTSD is a persistent disorder caused by pathological memories. This particular mechanism predicts that PTSD symptoms occur through the loss or enhancement of traumatic memories (Shackman et al., 2011; Näätänen et al., 2012). This finding suggests that changes in the brain in PTSD patients are based on the amygdala, medial prefrontal cortex, and hippocampus as the core (Shalev et al., 2017). However, we did not observe changes in the amygdala and hippocampus, probably because the observation period was short and changes in certain brain regions were insufficient to be detected.

Certain studies suggested that changes in the right SFG in PTSD could be related to altered emotional processing (Yin et al., 2012) and that social support and coping styles influenced the mood of individuals with PTSD. The total PCL-5 scores for objective support and subjective support showed a negative correlation in the study. Numerous studies have revealed an association between social support and the onset of PTSD (Breet et al., 2014). Traumatic events will cause the expression of negative emotions, and their persistent existence can damage the physical and mental health of the individual, resulting in PTSD (Zheng et al., 2012). Individuals with low social support have an increased risk of PTSD (Nhac-Vu et al., 2014). In addition, the coping style was reported to be associated with the onset of PTSD (Wu et al., 2011). In this study, the PCL-5 score was negatively correlated with positive coping. Individuals using negative coping styles received relatively less social support, and those using positive coping styles were more likely to receive social support. Low social support and constant stress increase the risk of developing PTSD, implying that good social support and positive coping styles after trauma could lower the risk of PTSD.

Recently, in addition to psychotherapy and medication, non-invasive neuromodulation techniques have been used as a new clinical intervention for PTSD. Common treatment modalities include transcranial direct current stimulation that induces action potentials in and around magnetically stimulated nerve tissue and repetitive transcranial magnetic stimulation that biases the cortical excitability of the area stimulated (Dayan et al., 2013). When selecting stimulation locations, previous studies have used dorsolateral prefrontal and medial prefrontal stimulation targets (Clark et al., 2015). Stimulation could be more effective if the target areas are those with abnormal brain activity in PTSD patients, which has been a popular area of research recently.

The limitation of this study is that the study population was from Shihezi and nearby areas, and the sample size was small, which could have impacted the results of this study. Secondly, only a small number of participants completed the third fMRI examination at 3 months, which could not be compared with the previous examination. The follow-up time in the later period was short, and the differences in the brain structure in other parts of certain patients could not be observed yet. Therefore, future studies require a larger sample size and long-term follow-up to obtain more valuable results.

## Conclusion

Despite the limitations, this longitudinally observed study showed that 2 months after MVA of PTSD patients had a reduced volume in the right SFG and left STG. In addition, PTSD patients received less social support and had negative coping styles. We believe the results of our study will provide useful insights into the neuropsychological mechanisms underlying PTSD.

## Data availability statement

The original contributions presented in this study are included in the article/supplementary material, further inquiries can be directed to the corresponding authors.

## Ethics statement

The studies involving human participants were reviewed and approved by the Ethics Committee of the First Affiliated Hospital of Shihezi University School of Medicine. The patients/participants provided their written informed consent to participate in this study.

## Author contributions

LY: data curation, formal analysis, original draft of the manuscript, and revision of the manuscript. HL and YM: data collection, data curation, and analysis. YS and AG: data analysis. GZ: revision of the original manuscript, project administration, supervision, and finalizing the manuscript. CL: revision of the manuscript. All authors contributed to the article and approved the submitted version.

## Footnotes

1
http://www.fil.ion.ucl.ac.uk/spm
